# Ice-Crystal-Templated “Accordion-Like” Cellulose Nanofiber/MXene Composite Aerogels for Sensitive Wearable Pressure Sensors

**DOI:** 10.1021/acssuschemeng.2c05597

**Published:** 2023-02-13

**Authors:** Wangwang Xu, Qinglin Wu, Jaegyoung Gwon, Jin-Woo Choi

**Affiliations:** † School of Renewable Natural Resources, Louisiana State University AgCenter, Baton Rouge, Louisiana 70803, United States; ‡ Forest Products Department, National Institute of Forest Science, 57 Hoegiro, Dongdaemun-gu, Seoul 02455, Korea; § Department of Electrical and Computer Engineering, Louisiana State University, Baton Rouge, Louisiana 70803, United States

**Keywords:** cellulose nanofibers, MXene, aerogels, “accordion-like” hierarchical architecture, strain sensing, biodegradation

## Abstract

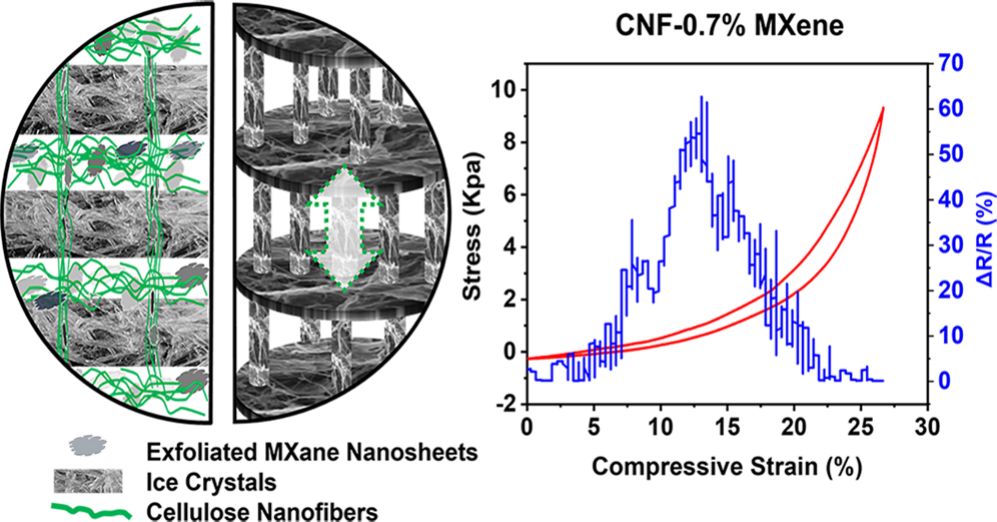

Exfoliated MXene nanosheets are integrated with cellulose nanofibers (CNFs) to form composite aerogels with high electric conductivity. The combination of CNFs and MXene nanosheets forms a unique “accordion-like” hierarchical architecture with MXene-CNF pillared layers through ice-crystal templating. Benefiting from the special “layer-strut” structure, the MXene/CNF composite aerogels have low density (50 mg/cm^3^), excellent compressibility and recoverability, as well as superior fatigue resistance (up to 1000 cycles). When being used as a piezoresistive sensor, the composite aerogel exhibits high sensitivity upon different strains, stable sensing performance with various compressive frequencies, broad detection range, and quick responsiveness (0.48 s). Moreover, the piezoresistive sensors are shown to have an excellent real-time sensing ability for human motions such as swallowing, arm bending, walking, and running. The composite aerogels also have a low environmental impact with the natural biodegradability of CNFs. The designed composite aerogels can serve as a promising sensing material for developing next-generation sustainable and wearable electronic devices.

## Abstract 2

Conductive CNF/MXene nanosheet composite aerogels were fabricated *via* ice-crystal templating through a freeze-drying process for next-generation wearable electronic devices.

## Introduction

The emergence of artificial intelligence has stimulated the rapid development of wearable sensor devices with high sensitivity, super lightweight, high stability, and excellent mechanical compliance.^1−5^ High-performance sensors present tremendous potential in broad applications such as sports monitoring devices, bionic limbs, and smart robots.^6−8^ Traditional sensors made of metals and inorganic semiconductors present good stability and high sensitivity.^9^ However, high costs and ultralow detection limits seriously hinder their applications. Therefore, finding an efficient and reliable wearable pressure sensor with low cost is challenging and meaningful.^6^

Based on the working mechanism, pressure sensors can be classified into capacitive, frictional, piezoelectric, and piezoresistive sensors.^4^ Piezoresistive sensors operate through changeable resistance *via* a controllable internal structure. Compared with other kinds of sensors, piezoresistive sensors are more widely applied in many fields due to their rapid response and flexible deformation. Currently, two strategies have been applied to improve the sensitivity and sensing range of strain sensors. One strategy is to manipulate the architecture of sensing materials, such as introducing arrays and wrinkles.^10^ Another strategy is to design some novel materials with high conductivity and tunable microarchitecture to improve their sensing prosperity.^11,12^ Strain sensors based on one-dimensional (1D) materials generally provide a broad sensing range, but they generally have poor sensitivity. This is attributed to the high aspect ratio of 1D materials, which leads to inconspicuous resistance changes under an extended strain range. Conversely, strain sensors based on two-dimensional (2D) materials usually show good sensitivity, but low stability.^13^ In recent years, strain sensors based on three-dimensional (3D) porous structures have shown an increased application potential due to their outstanding properties of high porosity, high stability, and high sensitivity.^14,15^ Aerogels with high conductivity have proven to be effective in the field of strain sensors.^16^ Therefore, a series of conductive aerogels with 3D structures such as polyurethane/carbon nanotube foams and polyimide/carbon nanotube aerogels have been developed and applied as pressure sensors for real-time monitoring.^17,18^

Carbon-based materials are the most common materials to fabricate conductive aerogels.^19^ For example, carbon aerogels can be synthesized by high-temperature pyrolysis (>1000 °C).^20^ The carbon aerogels attracted tremendous interest due to their outstanding properties such as large specific surface area, lightweight, and high sensing ability.^21,22^ However, the extreme preparation conditions of high pressure, high temperature, and oxygen-free environment seriously limit their large-scale production. In addition, high-temperature annealing often leads to low mechanical properties such as brittleness and poor compression resilience. Therefore, tremendous research interest has been devoted to the combination of conductive fillers and flexible matrixes to explore applicable and efficient strategies for piezoresistive sensors.^7^ Recently, a series of conductive fillers such as carbon nanotubes (CNTs), metal nanoparticles, graphene, and conductive polymers have been combined with various elastic substrates and show great potential applications in piezoresistive sensors.^23−27^ For example, Wu et al. developed composite strain sensors based on graphene aerogels and polydimethylsiloxane.^28^ The synthesized composite sensors showed a high sensitivity with a high gauge factor value of up to 61.3. Moreover, the freezing point can be adjusted using the concentration and cell size of graphene aerogels. Therefore, the designed strain sensors based on the composite materials are still workable at the freezing temperature of −196 °C. Han et al. designed hybrid elastomers based on cellulose nanofibers (CNFs) and polyaniline.^29^ The aniline monomers polymerize directly on the surface of CNFs, forming a hierarchical 3D network structure with electroconductivity. The high conductivity and sensitivity enable the hybrid elastomers to monitor real-time human motion. All of these designed strain sensors show good sensing ability and high stability. However, large residual deformation in multiple cycles, high cost, low biocompatibility, and poor detection capacity seriously restrict their practical applications.

In recent years, MXene, an emerging new two-dimensional material has attracted tremendous attention and is being applied in various fields.^30^ Generally, MXene is an early transition-metal carbide or carbonitride material with a graphene-like laminated structure.^31^ Until now, there are over 30 kinds of synthesized MXenes. Because of its excellent properties of high conductivity, mechanical stability, and numerous chemical groups on its surface, MXene has been widely studied in the fields of catalysts, batteries, and electromagnetic shielding.^30,32−34^ For example, Chen et al. prepared the Ti_3_C_2_T*_x_*/WSe_2_ hybrid materials as gas sensors with ultrafast response/recovery properties and low electrical noise.^35^ Because of the heterojunctions formed by Ti_3_C_2_T*_x_*/WSe_2_ nanohybrids, the sensitivity of the hybrid sensor is improved by over 12-fold compared with pristine Ti_3_C_2_T*_x_* and pristine WSe_2_. In the most recent years, MXene has been also adopted for the fabrication of strain sensors. For example, Yang et al. developed a Ti_3_C_2_T*_x_* MXene nanoparticle–nanosheet hybrid network as a strain sensor with high sensitivity and a broad testing range.^12^ The synergetic effect of nanoparticles and nanosheets endows the hybrid network with high electrical–mechanical ability. However, as strain sensing materials, pure MXene nanosheets have challenges of limited stretching range due to the rapid crack propagation of stacked MXene sheets, which seriously hinders the application of MXene-based sensors.

As one of the most abundant natural polymers, CNFs are widely applied as renewable reinforcing additives in various composites due to their properties of high mechanical strength, low cost, and natural friendliness.^36^ Inheriting the merits of CNFs, CNF aerogels generated by ice templating through freeze-drying maintain excellent mechanical stability.^36,37^ However, due to the nonconductive nature of CNFs, CNF aerogels have limited application potential as piezoresistive sensors.^38^ Modification of the MXene surface to have numerous hydroxyl groups similar to those on CNFs enables the firm chemical bonding between MXene nanosheets and CNFs. Therefore, combining conductive MXene nanosheets with CNF aerogels forms a promising strategy for designing new strain sensors.

Herein, conductive CNF/MXene nanosheet composite aerogels were fabricated *via* ice-crystal templating through a freeze-drying process. In particular, the entangled CNF framework serves as a skeleton for the whole composite aerogel with remarkable mechanical stability. MXene nanosheets provide high electrical conductivity. The morphology, microstructure, formation mechanism, electrical properties, and sensing performance were systemically investigated. The special “layer-strut” cellular structure endows an integrated composite with high electrical conductivity, low density, excellent compressibility and recoverability, and superior fatigue resistance. As a piezoresistive sensor, the CNF/MXene composite aerogels exhibit a high sensing ability at different strains and a durable sensing ability. Moreover, the piezoresistive sensors based on CNF/MXene composite aerogels were also applied to monitor real-time human motions, including swallowing, arm bending, walking, and running. Unlike commercial synthetic sponges and rubbers, the prepared MXene/CNF aerogels can be naturally biodegraded in soil, showing an attractive close-loop recycling feature. As a multifunctional piezoresistive sensor, the MXene/CNF aerogel sensor achieves remarkable mechanical properties, sensitive sensing ability, and sustainable features of CNFs with great application potential in human motion monitoring.

## Experimental Section

### Chemical Treatment for CNFs

A stable uniform CNF suspension was formed using a chemical treating method.^39^ First, 2.1 g of NaOH and 3.6 g of urea were added into 20 mL of deionized water (28.5% total chemical concentration). After NaOH and urea were fully dissolved, 1.8 g of CNFs (15 wt % water suspension, University of Maine, Orono, ME) were dispersed into the solution under stirring at room temperature. Then, 40 mL of ethanol (99.9%) was added to the mixture, and the reaction was continued for 4 h. The treated CNFs were finally collected by centrifuging and washed with water and ethanol several times until pH 7 was reached.

### Preparation of Exfoliated Ti_3_C_2_T*_x_* MXene Nanosheets

Exfoliated Ti_3_C_2_T*_x_* MXene nanosheets were synthesized based on the method described in previously published literature studies.^40−42^ First, 2 g of lithium fluoride (LiF) powder was slowly added to 20 mL of 12 M HCl solution under continuous stirring. After the LiF powder was dissolved completely, 1 g of Ti_3_AlC_2_ powder was slowly added to the solution. The mixture was then continuously stirred at 45 °C for 24 h. Afterward, the solid residue was collected by centrifuging and washed with 1 M HCl solution several times to remove the residual HF. Next, the solid residue was washed with deionized water several times until the pH value reached 6.0. Subsequently, the collected solid residue was dispersed in 200 mL of deionized water and ultrasonicated for 2 h under an argon atmosphere in an ice bath. Finally, the exfoliated MXene nanosheet suspension was collected by centrifuging at 3500 rpm for 1 h. The concentration of MXene was about 2 mg/mL in the suspension.

### Fabrication of CNF/MXene Composite Aerogels

The prepared CNF suspension was diluted to 0.8 mg/mL by adding deionized water. The volume of the prepared CNF suspension was around 3 mL. Then, 0.5 mL of the prepared exfoliated MXene suspension was added to the suspension. After being stirred for half an hour, the mixture formed a uniform wet gel. The formed wet gel was then transferred to a plastic mold and placed in a freezer at −80 °C for 12 h. Finally, the CNF/MXene composite aerogels were obtained by freeze-drying for three days.

### Material Characterization

X-ray diffraction (XRD) spectra of all of the samples were acquired using a Rigaku MiniFlex XRD instrument (Rigaku, Austin, TX) with Cu Kα radiation (λ = 1.5405 Å) at a working current of 40 mA and voltage of 40 KV from the 2θ range of 5 to 90° (1° per minute scan rate). To investigate the chemical state of MXene before and after exfoliation, X-ray photoelectron spectroscopy (XPS) spectra were collected on an AXIS165 spectrometer (MRFN, Manchester, U.K.). The microstructure of MXene nanosheets, CNF aerogels, and CNF/MXene composites (sprayed-coated with platinum for 2 min) was observed using an FEI Quanta 3D FEG field emission scanning electron microscope (SEM) (FEI, Boston, MA) at an acceleration voltage of 20 kV. Energy-dispersive spectroscopy (EDS) mapping was also conducted with the same SEM instrument. A Thermo Scientific Nicolet 6700 (Waltham, Boston, MA) spectrometer was used to collect Fourier transform infrared (FTIR) spectra in the spectral range from 400 to 4000 cm^–1^ for pure CNF aerogels, exfoliated MXene nanosheets, and CNF/MXene composite aerogels. The morphologies and crystallographic structures of all of the samples were further investigated with a high-resolution transmission electron microscope (HRTEM)—a JEM-1400 (JEOL USA Inc., Peabody, MA). Thermogravimetric analysis (TGA) was performed using a Q50 analyzer (TA Instruments Inc, New Castle, DE) with a 1 °C min^–1^ heating rate from 30 to 800 °C under a nitrogen atmosphere. The mechanical properties of the CNF/MXene composite aerogels were measured using a Model 5900R Instron machine (Instron Inc., Norwood, MA) equipped with an 890 N load cell. The diameter of the cylindrical samples for strain response testing was about 20 mm, and the height was about 5 mm. The piezoresistive sensing performance was measured using an eight-channel LAND battery analyzer (CT3001A, LAND Electronics Corporation, Wuhan, China). A conductive tape was attached to both sides of the composite aerogel to the analyzers to obtain the output of electrical signals.

The biodegradation test was performed according to a standard anaerobic biodegradation method (American Wood Preservation Association—AWPA E10—Laboratory Method for Evaluating the Decay Resistance of Wood-based Materials against Pure Basidiomycete Culture: Soil Block Test). Each selected plastic container (110 mm in diameter and 80 mm in height) was filled with a target weight of preprepared forest soil. Water was added to each container to reach the soil’s water-holding capacity. Two wood feeder strips were placed on the top of the soil in each container. A brown fungus (i.e., *Postia placenta*) was first grown on agar media in Petri dishes for two weeks. Fungus inocula were cut into 10 mm circular plugs from the growing edge of the Petri dish culture and placed on top of the soil close to each feeder strip in each container. After a two-week growing period (allowing the fungi to grow on the feeder strip), three groups of samples (i.e., polyurethane sponge as the control sample, pure CNF aerogels, and the prepared CNF/MXene composite aerogels) were placed on the top of the feeder strips in each container. These prepared plastic containers were kept in a conditioning chamber set at a temperature of 25 °C and relative humidity of 85%. After four months, the samples were taken out from test containers for morphology and weight loss analysis.

## Results and Discussion

### Basic Properties of the CNFs and MXene for Composite Aerogels

**Figure 1:**
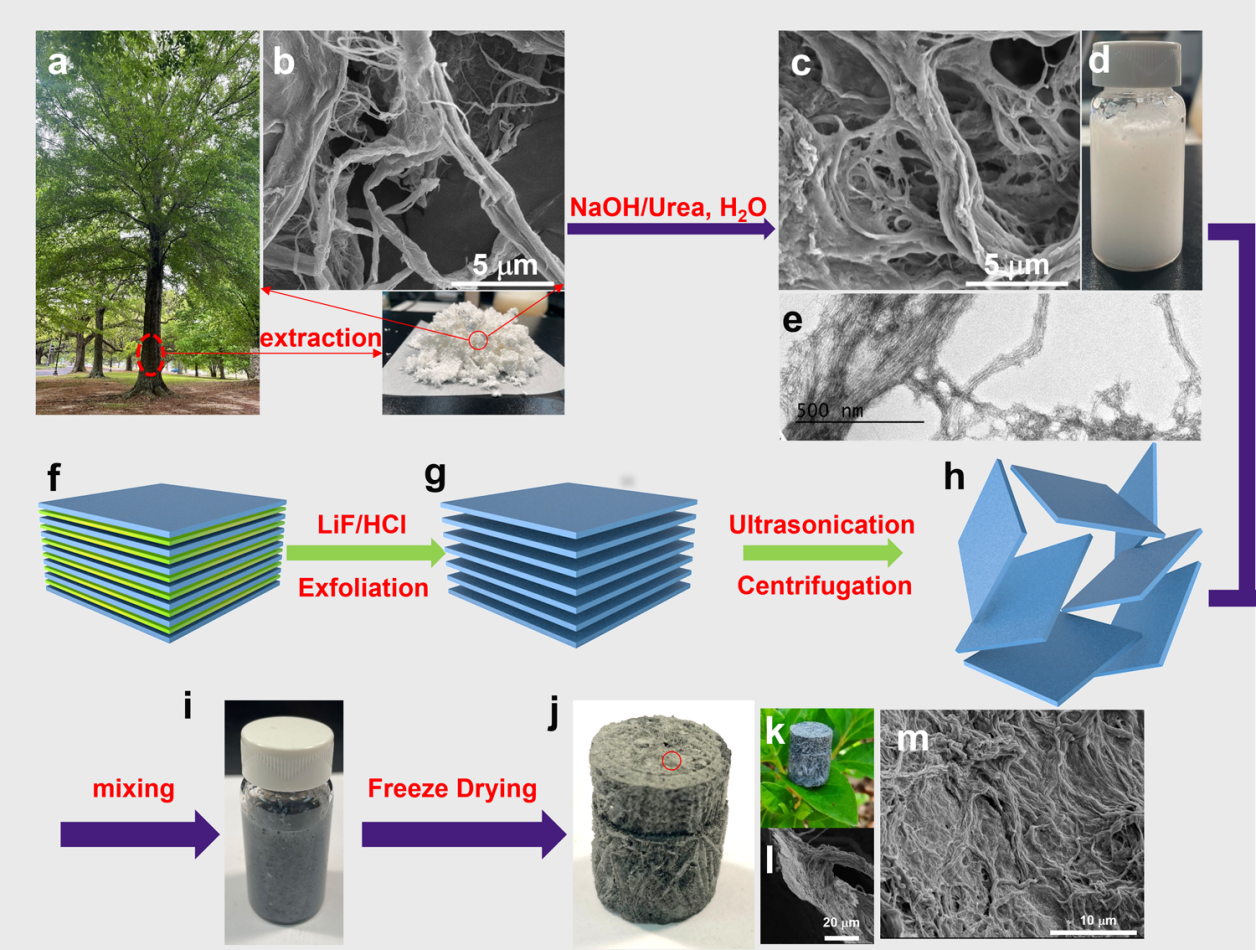
Schematic illustration of the procedure for the preparation of CNF suspension, exfoliated MXene nanosheets, and CNFs/MXene composite aerogels: (a) a tree, (b) CNFs extracted from wood (inset is the photo of the CNF powder), (c) chemically modified CNFs, (d) the photo of the CNF suspension, and (e) TEM image of CNFs after chemical treatment. (f–h) Schematic illustration of the exfoliation process of MXene, (i) optical images of the CNF/MXene composite suspension, (j, k) optical images, and (l, m) SEM images of the surface of the prepared CNF/MXene composite aerogels.

Commercial CNFs extracted from wood were used as the starting material (Figure 1a). The material had both individualized fibers and largely entangled fiber bundles. It is known that sodium hydroxide and urea can penetrate into interfibril regions of fiber bundles and separate the entangled microfibrils to form more individualized fibrils. Specifically, sodium hydroxide and urea system cleaves intramolecular hydrogen-bonding interactions in CNFs, leading to the rearrangement of molecular chains, significant fiber swelling, and separation.^43^ After being washed with deionized water and the pH value was adjusted to 7, partial hydrogen bonds were reconstructed on the surface of individual CNFs due to the protonation effect.

TEM images were obtained to investigate the morphology and microstructure of CNFs before and after alkali/urea treatment. As shown in Figure S1a, the highly entangled CNFs (diameter: 32 nm) before the chemical treatment were broken down to form more individual fibers (diameter: 10 nm) and bundles with reduced diameters after the treatment (Figure S1b). During the chemical treatment of CNFs, the sodium hydroxide and urea system cleaves intramolecular hydrogen-bonding interactions in CNFs, leading to the rearrangement of molecular chains, significant fiber swelling, and separation.^43^ After being washed with deionized water and the pH value was adjusted to 7, partial hydrogen bonds were reconstructed on the surface of individual CNFs due to the protonation effect. The processed CNF suspension can serve as an ideal dispersant for MXene nanosheets, forming a uniformly dispersed CNF and MXene mixture in water.

The exfoliation procedure of MXene (Ti_3_C_2_T*_x_*) from Ti_3_AlC_2_ is illustrated in Figure 1b. The LiF/HCl hybrid solution was first used to delaminate the Ti_3_AlC_2_ precursor by selectively removing the aluminum layers. At the same time, a series of polar functional groups were formed at the surface of MXene. After the chemical etching, MXene formed an architecture similar to an accordion, which is shown in Figure S2a,b. A subsequent ultrasonic exfoliation procedure helped further peel off the MXene into monolayers.

**Figure 2:**
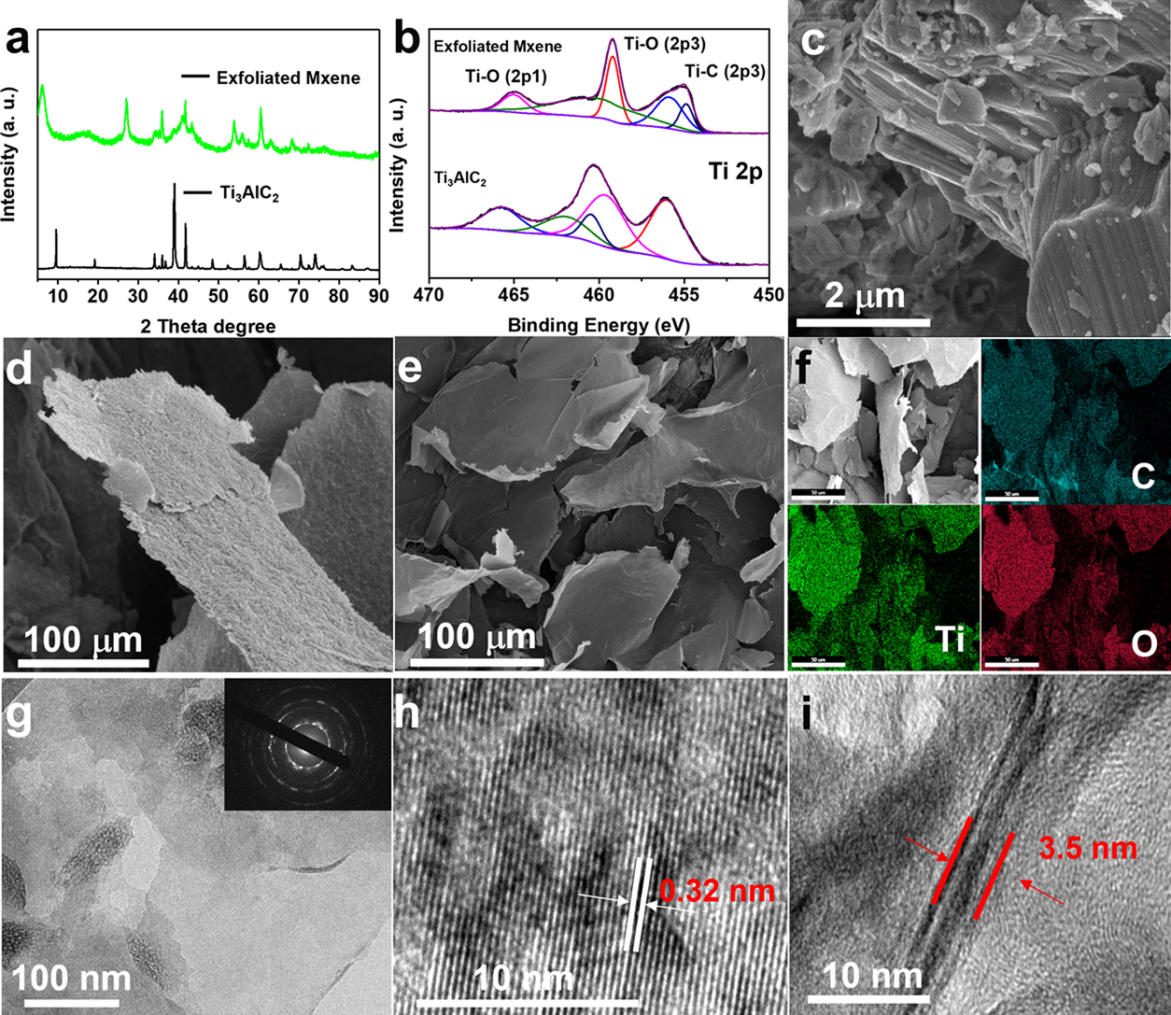
Material characterization data of MXene before and after chemical exfoliation. (a) XRD patterns and (b) XPS spectra of pristine Ti_3_AlC_2_ and exfoliated MXene nanosheets. (c) SEM images of pristine Ti_3_AlC_2_. (d, e) SEM images, (f) EDS mapping, (g) TEM images (SAED inset), and (h, i) HRTEM images of exfoliated MXene nanosheets.

The XRD patterns of the pristine Ti_3_AlC_2_ precursor and exfoliated MXene nanosheets are shown in Figure 2a. Compared with the pristine Ti_3_AlC_2_ precursor, the (002) peak of exfoliated MXene nanosheets downshifts from 9.66° to 6.46°, and the (104) peak at 39.04° is much weakened, which is similar to the results in the previously published reports, revealing the successful exfoliation of MXene nanosheets.^41^ XPS results before and after exfoliation are shown in Figures 2b, S2, and S3. The peaks of Ti 2p and C 1s spectra confirm the existence of Ti–C and Ti–O bonds, suggesting the formation of Ti_3_C_2_(OH)_2_ after exfoliation.^44^ The results are in an agreement with the findings in the previously published literature.^45^ SEM images of the pristine Ti_3_AlC_2_ precursor and exfoliated MXene nanosheets are shown in Figure 2c–e. As shown in Figure 2c, the pristine Ti_3_AlC_2_ precursor presents a bulk morphology with a random shape and rough surface. After the exfoliation treatment, the MXene shows a uniform thin nanosheet morphology with a smooth surface. The thickness of MXene nanosheets is only several nanometers, and the lateral size is around 100–500 μm. The EDS mapping in Figure 2f proves that C, F, Ti, and O elements are homogeneously distributed throughout the entire area, revealing the uniform composition of the exfoliated MXene nanosheets. TEM images in Figure 2g further confirm the small thickness of MXene nanosheets, which were almost semitransparent to TEM electrons. The selected area electron diffraction (SAED) pattern in the inset image of Figure 2g shows discontinuous ring patterns. Based on the calculation of the ring pattern, the hexagonal crystal structure of Ti_3_AlC_2_ is well maintained after exfoliation. Moreover, the HRTEM image in Figure 2g exhibits uniform parallel lattice fringes, suggesting the single-crystal nature of individual MXene nanosheets. In addition, the d-spacing of 0.32 nm corresponds to the (104) plane of the hexagonal MXene crystals. The HRTEM image in Figure 2i clearly shows three layers of the exfoliated MXene nanosheets. The spacing between layers was only 1.8 nm, which is consistent with the reported literature studies.^12^ These results intuitively confirm the successful exfoliation of the prepared MXene nanosheets.

### Microstructure of the Prepared CNF/MXene Composite Aerogels

**Figure 3:**
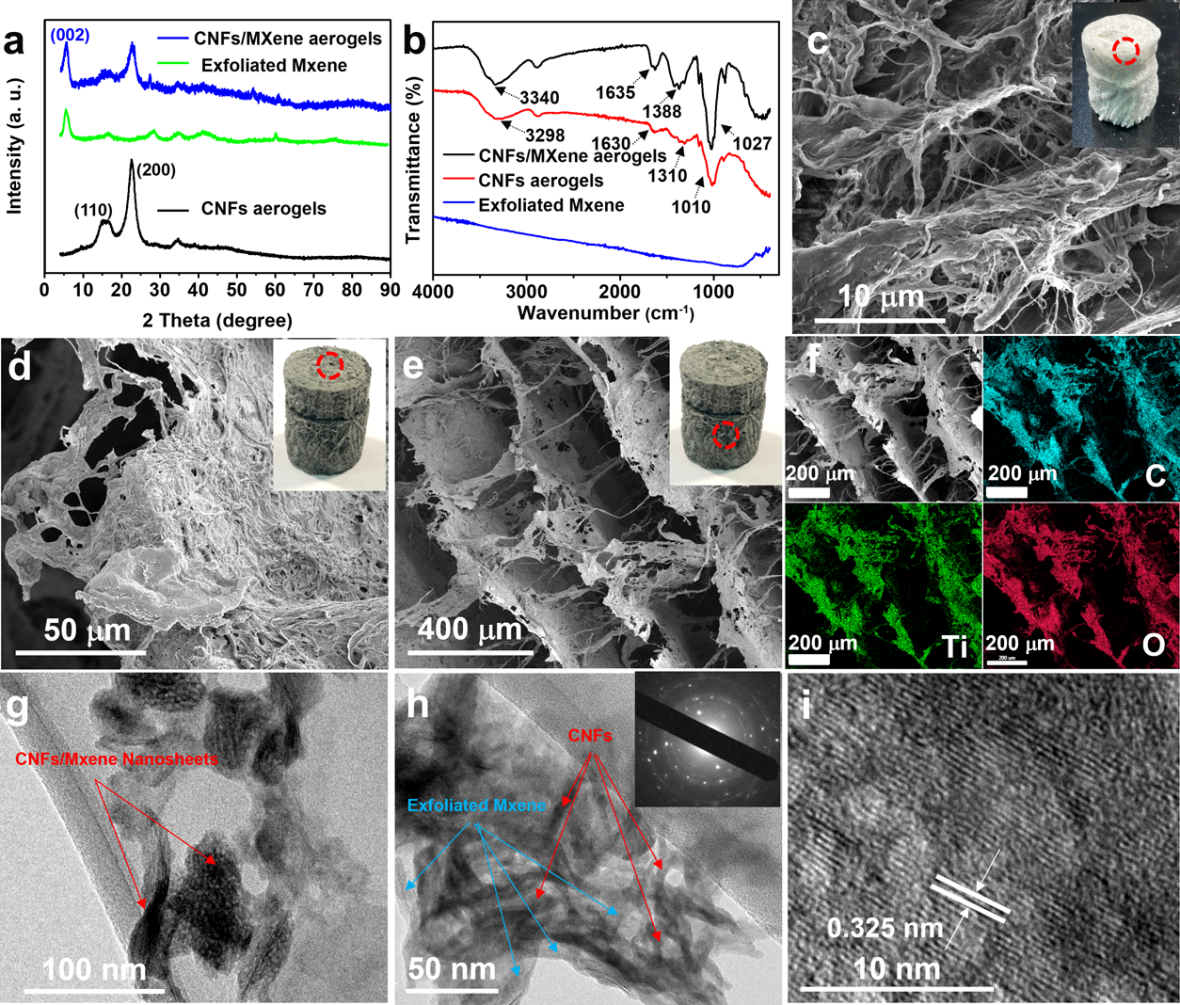
Material characterization of CNF/MXene composite aerogels. (a) XRD patterns and (b) FTIR spectra of pure CNF aerogels, exfoliated MXene nanosheets, and CNF/MXene composite aerogels. (c) SEM images of pure CNF aerogels. (d, e) SEM images, (f) EDS mapping, (g, h) TEM images (SAED inset), and (i) HRTEM images of CNF/MXene composite aerogels (3.0 wt % of MXene in the composite).

Ice templating through freeze-drying of CNFs and CNF/MXene mixture suspensions was successfully used to obtain CNF/MXene composite aerogels. Because of abundant hydrogen bonds on the surface of MXene nanosheets, a stable combination was formed between CNFs and MXene nanosheets. The crystal structures and functional groups on the composite aerogels were analyzed *via* XRD and FTIR techniques. As shown in Figure 3a, the CNFs after treatment showed a dominant cellulose I peak at about 22.5° (200) and two overlapped diffraction peaks at about 15.16° (11̅0) and 16.60° (110), presenting a typical cellulose I structure.^46^ After CNFs were combined with exfoliated MXene nanosheets, all of the characteristic peaks of cellulose I structure and the dominant diffraction peak at 6.46° from exfoliated MXene are present in the composite aerogel. Figure 3b shows the FTIR spectra of all three samples. The characteristic peak of 3340 cm^–1^ corresponds to the vibration of intermolecular −OH bonds, 2886 and 1364 cm^–1^ peaks are attributed to the stretching and bending vibration of C–H bonds, respectively, 1621 cm^–1^ peak corresponds to the stretching vibration of C=O double bonds, and the absorption peaks at 1020 and 890 cm^–1^ are ascribed to the pyranose ring skeletal vibration and stretching vibration of C–O–C bonds, respectively.^32^ The absorbance peaks at 545 cm^–1^ correspond to the terminal group of hydrogen bonds in MXene nanosheets. After the combination, no new peak is observed. A small red shift on 3340 and 890 cm^–1^ is attributed to the chemical bonding between MXene nanosheets and CNFs. The internal microstructures of the CNF/MXene composite aerogels are demonstrated in the SEM and TEM images. According to the inset images shown in Figure 3c, the CNF aerogels after chemical treatment display a pure white color with a low density of 50 mg/cm^3^. The CNF aerogels exhibit an isotropic open-cellular structure, composed of numerous entangled fibers. With an increase of the loading amount of MXene, the color of the composite aerogel changed from white to gray, as shown in Figures S4 and 3d.

The morphology of the CNF/MXene composites with different mixing ratios is displayed in Figure S4. Interestingly, the addition of MXene nanosheets had a significant impact on the microstructure of the prepared composite aerogels. As shown in Figure S4a–c, with the addition of only 0.7 wt % of MXene, the CNF/MXene composite formed a porous network structure composed of thin sheet films, which is similar to the structure of a honeycomb. With a further increase of the proportion of MXene up to 3.0 wt %, a well-ordered hierarchical laminated structure was formed. The composite aerogel had an obvious anisotropy structure. As shown in Figures 3d and S4d, the surface of aerogels showed a dense and flat CNF/MXene composite laminate with a lateral size of around 100–500 μm. The close-up views of a composite layer reveal that CNFs were firmly attached onto the surface of MXene nanosheets, forming an integrated composite laminate. As shown from the cross-sectional view, an “accordion-like” laminated structure was formed, where the composite nanofibers acted as a strut to connect adjacent CNF–MXene layers and the layer spacing is in the range of 100–200 μm (Figures 3e and S4e,f). The formation of this unique “layer-strut” bracing hierarchical nanofibrous structure is attributed to the ice crystal growth with an appropriate oriented direction during the freeze-drying process. During the ice-crystal growth, the delaminated CNF/MXene layers with composite fibers act as a strut to connect adjacent layers, forming a highly oriented structural integration. Magnified SEM images reveal that both the layers and the pillars are composed of CNFs and MXene nanosheets, which are firmly bonded together. The robust bonding between CNFs and MXene nanosheets can effectively improve their electrical conductivity and structural stability under high pressure. Moreover, the space between adjacent layers enables a large compression rate, and numerous pillars with high flexibility and bendability serve as the spring-back mechanism that can significantly enhance the recoverability. Besides, EDS mapping in Figure 3f demonstrates that all of the major elements including C, Ti, O, and F were uniformly distributed in both composite laminates and the pillar materials between them, proving the uniform composition throughout the entire aerogels.

A possible mechanism was proposed to explain the formation of this unique “accordion-like” CNF/MXene composite aerogels. Our early work demonstrated how the concentration of cellulose nanoparticles affected their self-assembling behavior during freeze-drying.^47^ In the present system, the unique “accordion-like” structure was formed due to the growth of ice crystals and their interaction with CNFs and MXene nanosheets.^48^ Before the freezing process (Figure S5), CNFs and exfoliated MXene nanosheets formed a steady and homogeneous suspension in water. After the suspension was frozen, ice crystals grew in the direction along the temperature gradient, creating a lamellar microstructure parallel to the direction of the movement.^49^ Meanwhile, CNFs and MXene nanosheets were firmly bonded together due to the van der Waals forces and hydrogen bonds, squeezed between ice dendrites, and self-assembled into the “accordion-like” structure.^3,49^ When the freezing process was completed, the initial uniform suspension system was completely transformed into the ice-crystal-templated structure. During the freeze-drying process, water molecules directly sublimated from the ice, generating a replica of the ice template, and finally forming the “accordion-like” structure. According to the SEM images shown in Figure S5, the CNFs and MXene nanosheets were compactly rearranged and self-assembled into an integrated “layer-strut” bracing structure.

To investigate the microstructure and crystallographic structure of the synthesized composite aerogels, TEM and HRTEM images were obtained. The TEM images in Figure 3g further confirm that CNFs and MXene nanosheets formed a uniform composite layered structure. Numerous pores in the composite nanosheets are attributed to the interfiber spaces in the entangled CNFs. No individual CNFs can be observed in both SEM and TEM results, further implying the strong bonding between CNFs and MXene nanosheets. As clearly shown in the TEM image in Figure 3h, the CNFs were firmly attached onto the MXene nanosheets. The MXene nanosheets also served as the platform for the distribution of all CNFs. The SAED inset in Figure 3h clearly presents the combination of ring pattern and scattered spot pattern, corresponding to the amorphous CNFs and crystal MXene nanosheets. In addition, the HRTEM image in Figure 3i confirms a d-spacing of 0.32 nm in the lattice fringes, suggesting that the hexagonal MXene crystals are well maintained in the composite aerogels.

### Electrical and Mechanical Properties of the CNF/MXene Composite Aerogels

**Figure 4:**
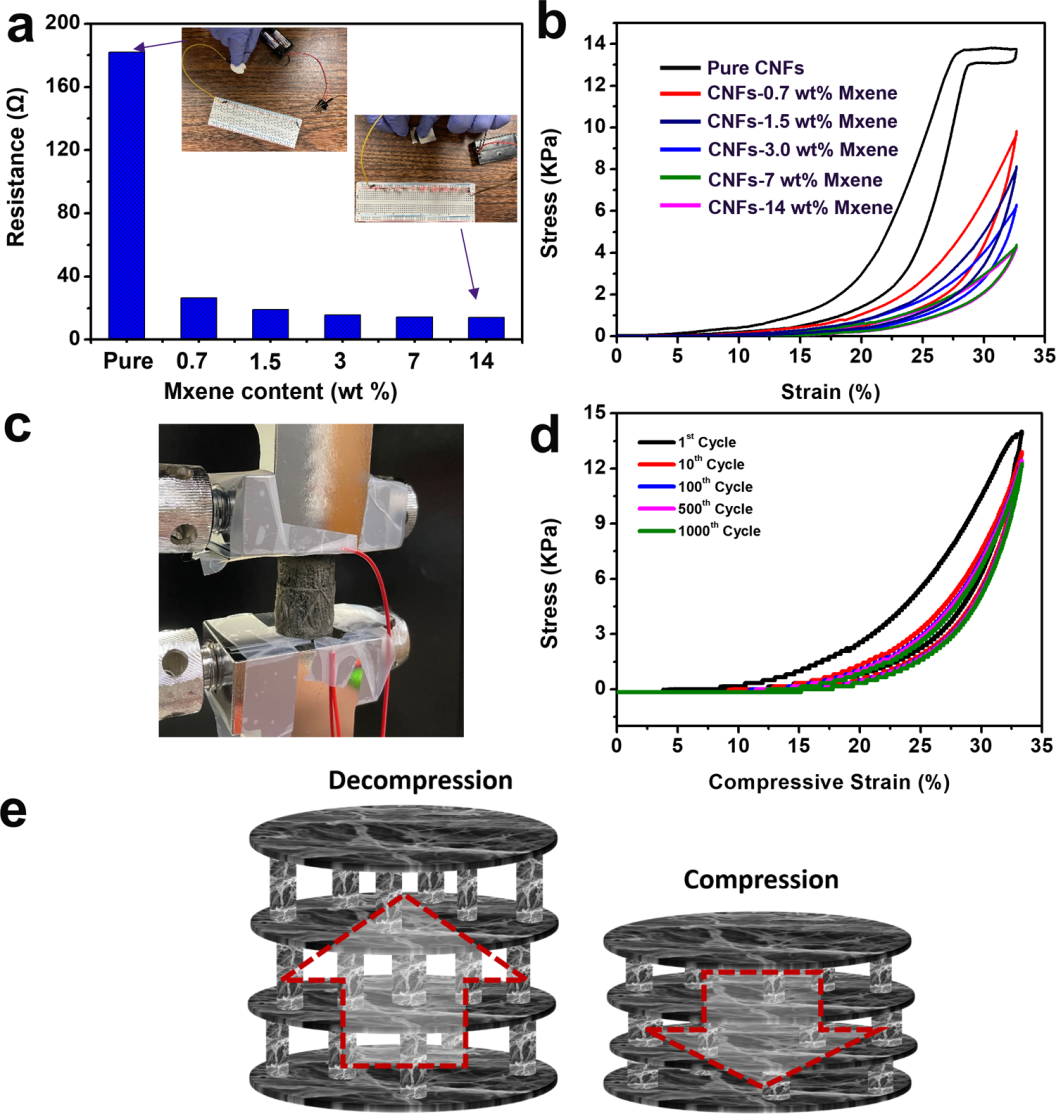
Mechanical properties of CNF/MXene composite aerogels. (a) Electrical conductivity testing for CNF/MXene composite aerogels with different ratios; (b) stress–strain curves of CNF/MXene composite aerogels with different ratios; (c) an optical image of the device with real-time sensing property reacting to pressure; (d) stress–strain curves of CNF/MXene composite aerogels for over 1000 cycles; and (e) schematic illustration of cyclic compression.

The electrical conductivity and mechanical properties of the prepared CNF/MXene composite aerogels with different mass ratios are depicted in Figure 4a. For the test samples of 20 mm in diameter and 5 mm in height, pure CNF aerogels showed a high electric resistance of about 182.0 Ω. The resistance of CNF/MXene composite aerogels decreased dramatically to 26.3 Ω with only 0.7 wt % of MXene added. Then, with the increased content of MXene, the resistance of composite aerogels changed slightly and decreased to 13.8 Ω with 14 wt % MXene in the composite. To verify the decrease of electrical resistance, LED lights were connected to the prepared aerogels. The LED lights became bright successfully after being connected with all of the composite aerogels, showing an improved electrical conductivity. The pure CNF aerogels, however, could not light the LED lights due to high resistance. The conductive nature of composite aerogels is the fundamental factor for resistive strain sensing properties. Mechanical properties of all of the prepared CNF/MXene composite aerogels are displayed in Figure 4b–d. With the stress–strain curves shown in Figure 4b, compared with pure CNF aerogels, the compressive strengths of the CNF–0.7 wt % MXene samples sharply dropped by 30% to 9.6 kPa. With an increased mass ratio of MXene, there was an obvious downward trend in stress at the same strain of 33%. In the three-dimensional network of CNF aerogels, there are numerous directional hydrogen bonds between the molecular chains, leading to increased compressive strength up to 13.8 kPa. The hydrogen bonding was much weakened between CNFs with the addition of MXene. Therefore, with the increased proportion of MXene, a gradual drop tendency was observed. Similar to the compressive strength, Young’s modulus decreased with increased MXene. Though the results demonstrated the negative impact of MXene on the strength of composite aerogels, the combination of MXene and CNFs led to a better internal architecture to withstand the destructive influence under pressure. Figure 4d shows the result of the compression resilience test for CNF/MXene composite aerogels. Satisfactorily, after 1000 cycles of repeated compression and decompression, the aerogels recovered 88% of the strength from the initial cycle at the same strain level of 33%. Figure 4e shows a schematic diagram of compression. The sufficient space between adjacent layers in the 3D “accordion-like” hierarchical structure can help release the deformations and external forces. Numerous pillars with high flexibility and bendability serve as springs to sustain strength and intrinsic power. The remarkable compressive resilience accompanying strain-dependent resistance satisfies the requirement for a piezoresistive sensor well.

**Figure 5:**
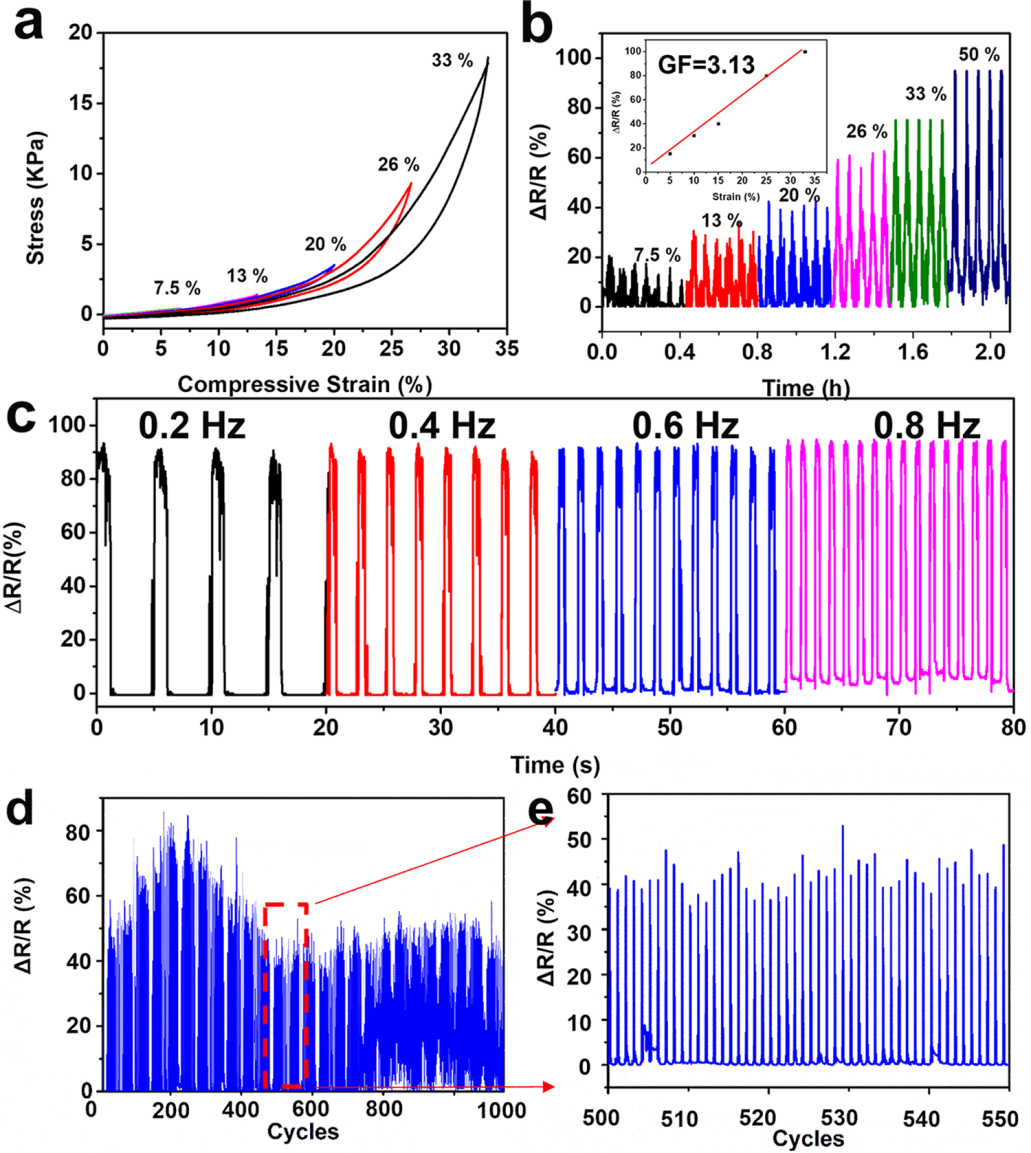
Piezoresistance property of CNF–3.0 wt % MXene composite aerogels. (a) Stress–strain curves at different strains; (b) related real-time sensing property reacting to different strains; (c) relative resistance change at different compression rates; (d) real-time sensing stability under repeated compression up to 1000 cycles with a strain of 33%; and (e) enlarged figure corresponding to panel (d).

Considering strain-dependent resistance, moderate mechanical compressive strength, and excellent recyclable compressibility, CNFs-3.0 wt % MXene composite aerogels were chosen as the study aerogel sensor. The piezoresistive sensing properties of the CNFs-3.0 wt % MXene composite aerogels were systematically investigated, and the results are presented in Figure 5. Figure 5a shows the stress–strain curves of the composite aerogels at different compressive strains. Related real-time sensing performance at different strains is depicted in Figure 5b. As shown in Figure 5b, the promising composite aerogel sensor had a wide range of detection. At the maximum strain of 7.5, 13, 20, 26, 33, and 50%, the sensor delivered a clear and distinct response at each strain or pressure level, showing a high sensing ability. Obviously, there was a linear tendency between the relative resistance chance, Δ*R*/*R*_0_, and applied strain. To assess the sensing ability of the strain sensor, the gauge factor (GF) was calculated. GF is defined as Δ*R*/Δε*R*_0_, in which Δ*R* is the change of resistance, *R*_0_ is the original resistance, and Δε is the change of strain. As shown in the inset image in Figure 5b, GF data were plotted as the function of strain and the resistance change. The GF values are consistent over a broad range of strains, reaching up to 3.13, which is comparable to the values of other reported strain sensors.^3^

To investigate the sensing stability of the designed composite aerogels, we measured the relative resistance changes reacting to different frequencies of compression, controlled by the speed of compression. As displayed in Figure 5c, at the frequencies of 0.2, 0.4, 0.6, and 0.8 Hz, the value of relative resistance changes (Δ*R*/*R*_0_) remained stable without any decaying, demonstrating no energy loss and high compression resilience. To further study the ability of the strain sensors during the long cycles of compression, the current change was tested with 1000 cycles of compression and decompression between the strain of 0 and 33% at a speed of 2 mm/s. As observed from Figure 5d, all of the current peaks maintained stable during these long-term cycles. From the magnified figure shown in Figure 5e, the individual sensing curves coincided well with each other, showing excellent long-term stability and monotonicity. Conclusively, the prepared CNF–3.0 wt % MXene composite aerogels present a highly sensitive sensing property, showing a great potential to detect fast-changing compressive behaviors.

### Real-Time Motion Sensing Ability of the CNF/MXene Composite Aerogels

**Figure 6:**
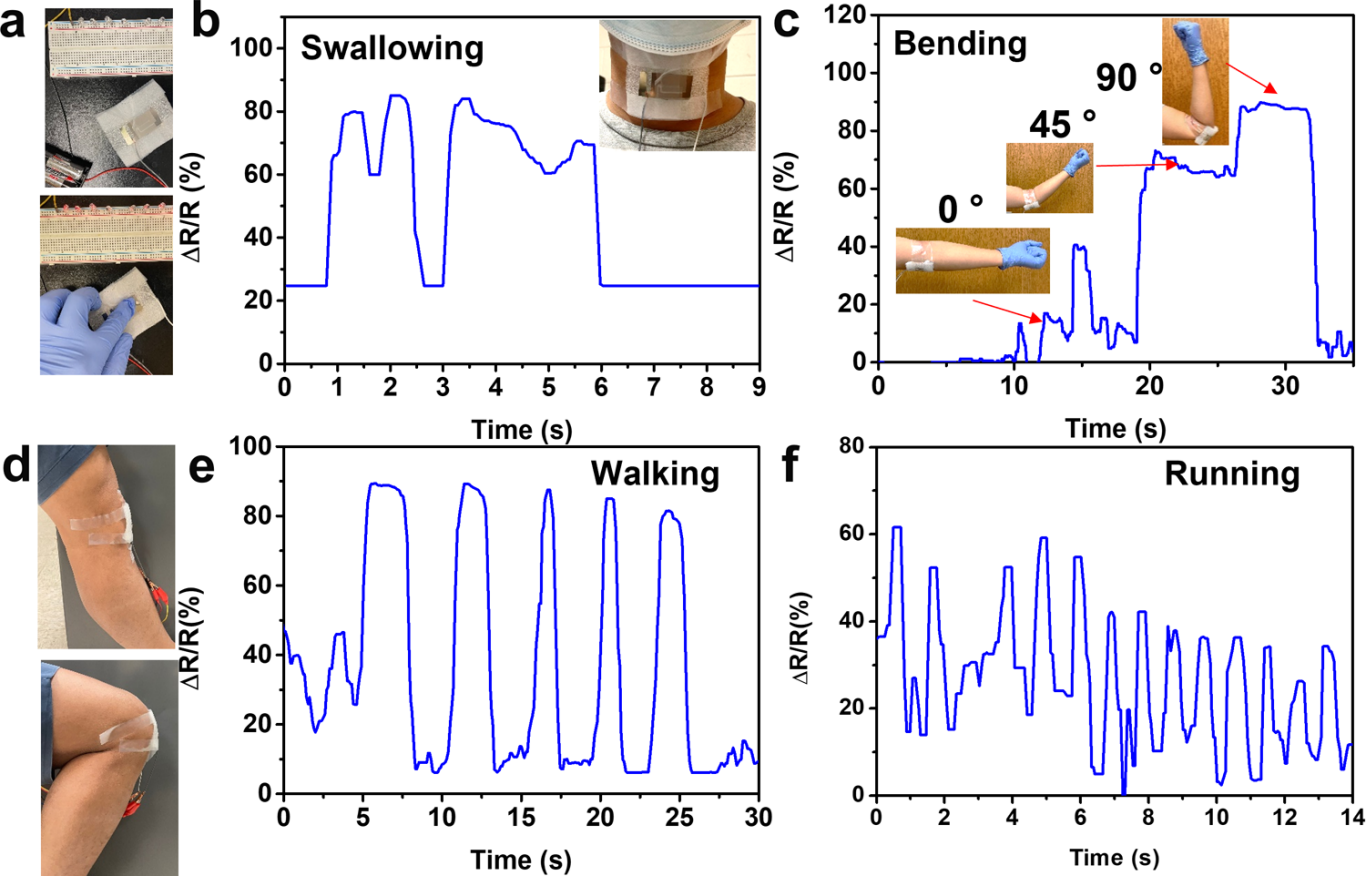
Real-time sensing performances of the CNF–3.0 wt % MXene composite aerogel served as a pressure sensor in monitoring human behavior while being adhered to the throat, an elbow, and a knee. (a) Red LED indicators connected with a pressure sensor before and after compression. Relative resistance changes of monitoring the behavior of (b) swallowing and (c) arm bending. (d) Optical image of attaching a designed strain sensor on a knee. Relative resistance changes of monitoring the behavior of (e) walking and (f) running.

The CNF–3.0 wt % MXene composite aerogel sensor demonstrates an outstanding sensing property, a wide detecting range, and excellent durability, which enables its practical application. As shown in Figure 6a, the composite aerogel sensor was designed as a switch for a row of LED indicators. The LED indicators were successfully lit up when the strain sensor was pressed. Moreover, as observed from the video in Supporting Information II, the LED indicators became much brighter when the strain sensor was further compressed. This is attributed to the significantly reduced resistance under compression. In addition, the strain sensor was further applied as a wearable device to detect a series of human motions. As displayed in Figure 6b, the strain sensor is capable of recording the relative resistance change upon throat swallowing. Besides, as shown in Figure 6c, the sensor attached to an elbow enables detection of the arm bending at different angles. At the bending angle of 0, 45, and 90°, the relative resistance change was proportional to the bending angles. When the strain sensor is attached to a knee, it can be used to monitor human’s motions (Figure 6d). Figure 6e illustrates the relative resistance change while the designed strain sensor was applied to monitor normal human’s walking. The composite aerogel sensor worked normally. It continuously captured the signals of each step while walking. Furthermore, the designed composite aerogel sensor was used to monitor fast running. As demonstrated in Figure 6f, the sensor detected the change of speed and pause during running. The little differences between each peak were generated by the diversity of movement, suggesting the high sensing ability of real-time detection. In addition, as it can be clearly observed from Figure 6f, the real-time response time for running can be as fast as 0.48, 0.79, and 0.9 s. The strain sensor can capture such a fast single, presenting excellent compressive resilience. Consequentially, this demonstration confirms that the wearable piezoresistive sensor based on CNF/MXene composite aerogels can be used to continuously monitor a full range of human motions, showing a potential application in wearable devices and electronic skins.

### Biodegradability of the Prepared CNF/MXene Composite Aerogels

**Figure 7:**
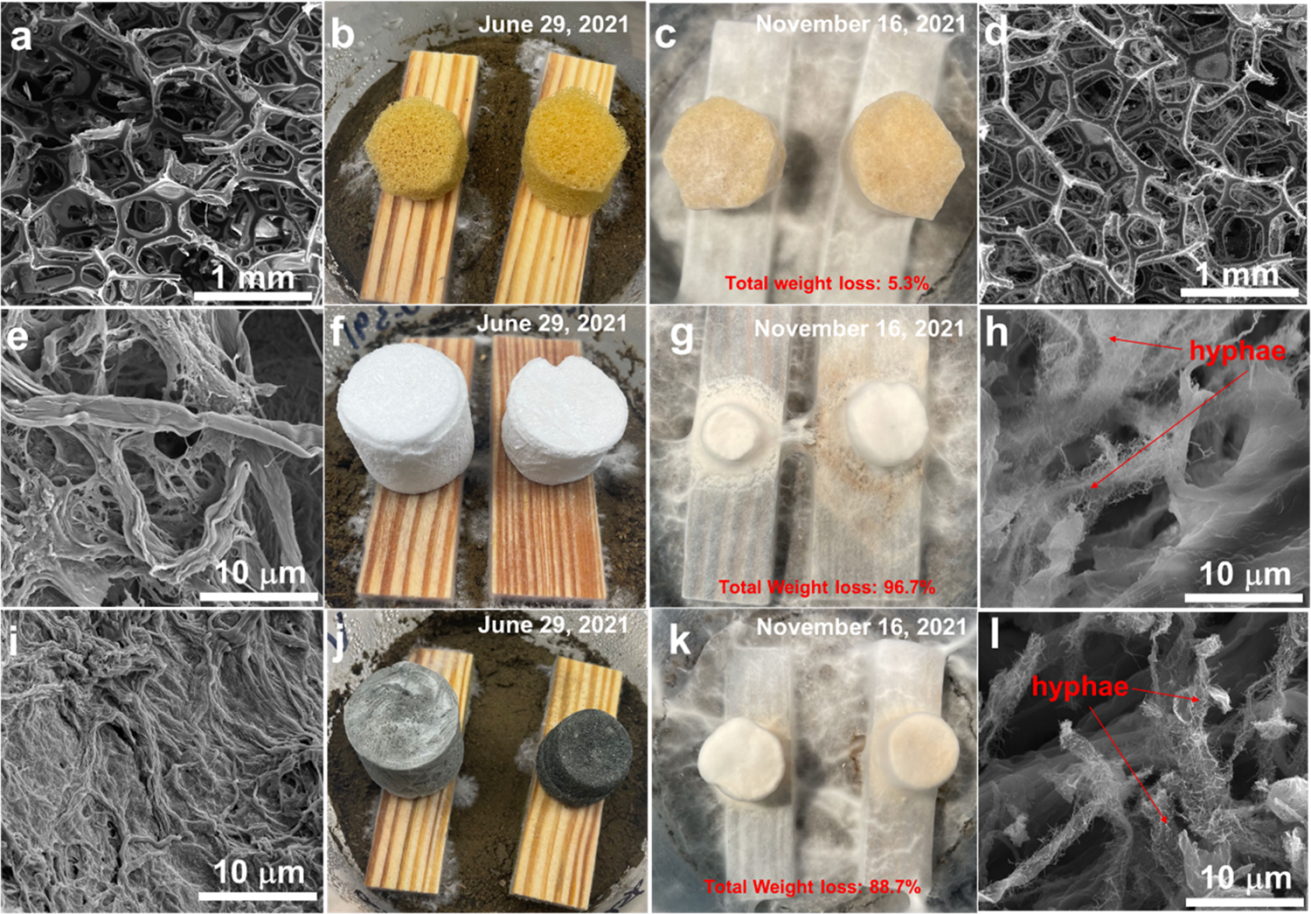
Biodegradability testing of CNF aerogels, CNF/MXene composite aerogels, and synthetic polymer sponges. SEM and digital images of (a, b) synthetic polymer sponges, (e, f) pure CNF aerogels, and (i, j) prepared CNF/MXene composite aerogels at the original state. SEM and digital images of (c, d) synthetic polymer sponges, (g, h) pure CNF aerogels, and (k, l) the prepared CNF/MXene composite aerogels after being biodegraded for 140 days.

A standard biodegradation test was applied to evaluate the degradability of the aerogels. After two months of testing, numerous fungus hyphae were observed in all containers, showing the growth of the test fungi (Figure S6). The CNF/MXene composite aerogels changed to white color owing to the oxidization of MXene and the formation of TiO_2_. Apparently, pure CNF aerogels and CNF/MXene composite aerogels became smaller in size after 2 months of biodegradation by fungi (Figure 7f,g,j,k). Fungi directly attacked and degraded the cellulose (i.e., CNFs) in the aerogels. Eventually, it became completely biodegraded after 5 months with a weight loss of 96.7% for pure CNF aerogels and 88.7% for the CNF/MXene composite aerogels. The degraded aerogel morphology was observed *via* SEM images. As shown in Figure 7e,h,i,l, the composite structure in pure CNF aerogels and CNF/MXene composite aerogels was severely changed by fungi. Continuous CNF/MXene laminates were fragmented, and long CNFs were broken. Numerous hyphae were found on the CNF surface, confirming the strong degradation. These prepared cellulose-based composite aerogels exhibit superior biodegradability in the natural environment. In contrast, the polyurethane sponge retained its original size and shape after the degradation for about 5 months with a weight loss of only 5.3% (Figure 7b,c). The SEM images in Figure 7a,d also confirm that the porous microstructure of polyurethane sponge maintained well after the degradation for about 5 months, suggesting the long-lasting degradation issue of the synthetic polymers on the environment. These prepared CNF/MXene composite aerogels are stable under working conditions but can be degraded under natural conditions, showing a great promise for next-generation sustainable and biodegradable materials.

## Conclusions

Conductive CNF/MXene composite aerogels with a unique “accordion-like” architecture were designed and fabricated *via* a controllable chemical treatment of CNFs, followed by a freeze-drying process. Because of the strong bonding between CNF and MXene nanosheets as well as the specific “layer-strut” bracing porous structure, the prepared composite aerogels exhibit high electrical conductivity, low density (50 mg/cm^3^), excellent compressibility and recoverability, and superior fatigue resistance (up to 1000 cycles). These merits enable the composite aerogels to serve as a sensitive piezoresistive sensor. The composite aerogel sensor shows a sensitive sensing ability upon compressions at different strains, stable piezoresistive sensing properties with various frequencies, broad detection range, and quick responsiveness (0.48 s). Moreover, the piezoresistive sensor based on composite aerogels can be applied to monitor real-time human motions such as swallowing, arm bending, walking, and running. The prepared CNF/MXene aerogels can be easily biodegraded by fungi in the soil through the degradation of CNFs, showing an attractive close-loop recycling feature. The designed CNF/MXene composite aerogels have remarkable mechanical properties, high piezoresistive sensing ability, and sustainable features, presenting great potential in applications for human motion monitoring. This strategy of fabricating “accordion-like” CNF/MXene composite aerogels helps create new opportunities for next-generation wearable electronics devices.

## Supporting Information Available

LED indicators were successfully lit up when the strain sensor was pressed (MOV)
EM micrograph of CNFs (Figure S1); FE-SEM micrograph of MXene (Figure S2); XPS spectrum of Ti3AlC2 (Figure S3) and exfoliated MXene (Figure S4); SEM images of the cross-section of CNF/MXene composite aerogels (Figure S5); schematic of the formation mechanism of the aerogels (Figure S6); and biodegradability testing pictures of the aerogels (Figure S7), and tabulated data for performance characteristics comparison of various materials (Table S1) (PDF)

## Supplementary Material

sc2c05597_si_001.mov
