# Supplementary material for: Ice-Crystal-Templated “Accordion-Like” Cellulose Nanofiber/MXene Composite Aerogels for Sensitive Wearable Pressure Sensors
Source: ACS Sustain Chem Eng. 2023 Feb 13;11(8):3208–18. doi: 10.1021/acssuschemeng.2c05597 (PMC9976353; doi:10.1021/acssuschemeng.2c05597)
Supplement: Supplementary file 2 — sc2c05597_si_002.pdf [file sc2c05597_si_002.pdf]

Supporting Information

Ice-crystal Templated "Accordion-Like" Cellulose Nanofiber / Mxene Composite Aerogels for Sensitive Wearable Pressure Sensors

Wangwang Xu[1], Qinglin Wu[1*], Jaegyoung Gwon[2*], and Jin-Woo Choi[3]

[1.] School of Renewable Natural Resources, Louisiana State University AgCenter, Baton Rouge, Louisiana 70803, United States

[2.] Forest Products Department, National Institute of Forest Science, 57 Hoegiro, Dongdaemun-gu, Seoul 02455, Korea

[3.] Department of Electrical and Computer Engineering, Louisiana State University, Baton Rouge, Louisiana 70803, United States

Corresponding email: wuqing@lsu.edu and gwonjg@korea.kr

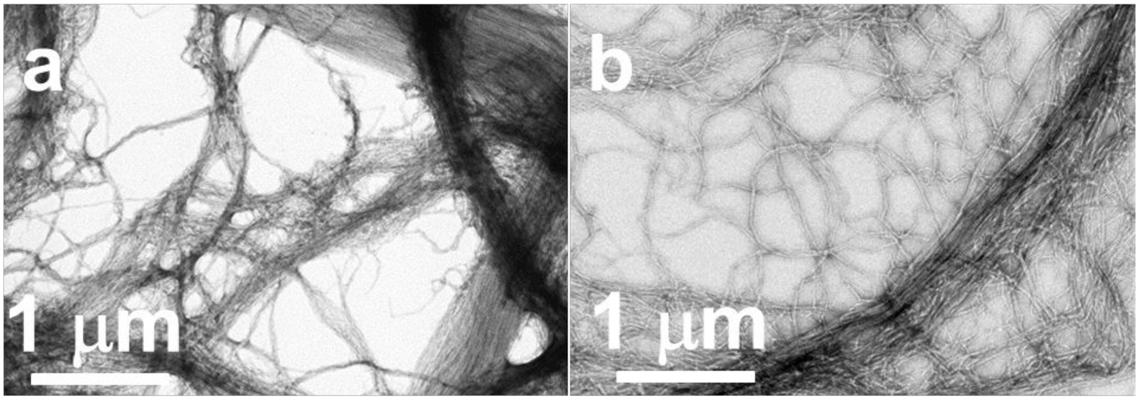

Figure S1. CNFs (a) before and (b) after chemical treatment. More individualized fibers were observed for treated CNFs.

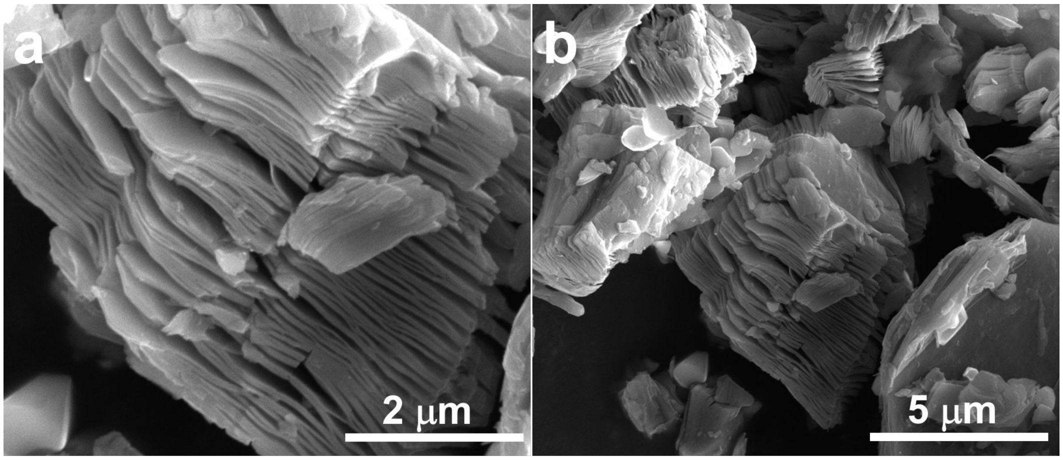

Figure S2. (a, b) SEM images of pristine MXene after chemical etching.

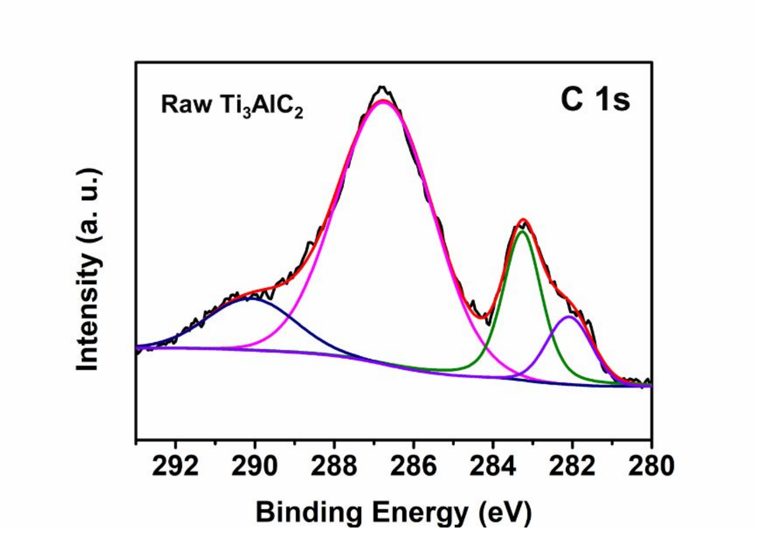

Figure S3. C 1s spectrum of Raw Ti3AlC2 sample

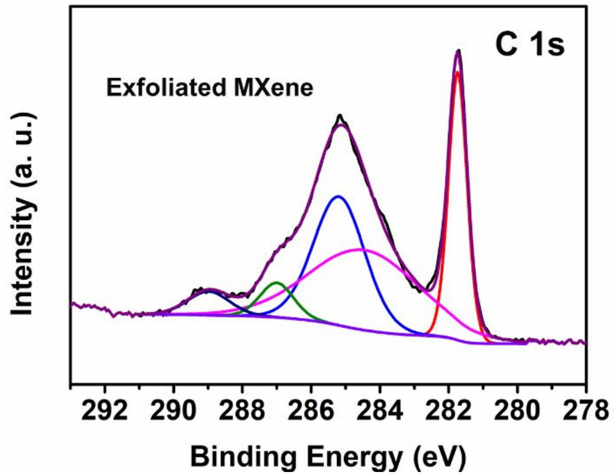

Figure S4. C 1s spectrum of exfoliated MXene sample

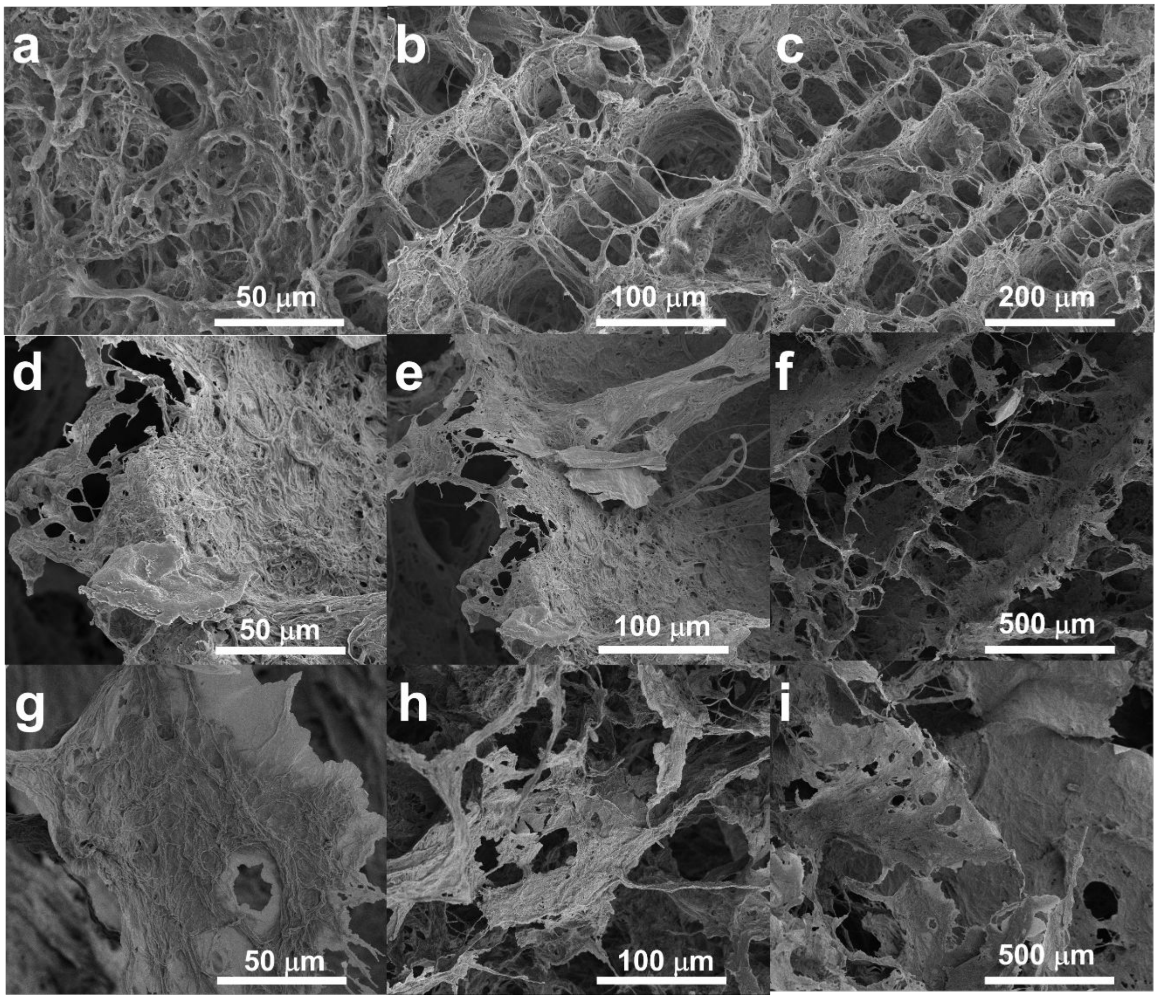

Figure S5. SEM images at cross-section of CNF/MXene composite aerogels with different material mixing ratios. SEM images of composite aerogels with 0.7 wt% of MXene at (a) surface and (b, c) cross-sections; SEM images of composite aerogels with 3.0 wt% of MXene at (d) surface and (e, f) cross-sections; SEM images of composite aerogels with 7.0 wt% of MXene at (g) surface and (h, i) cross-sections.

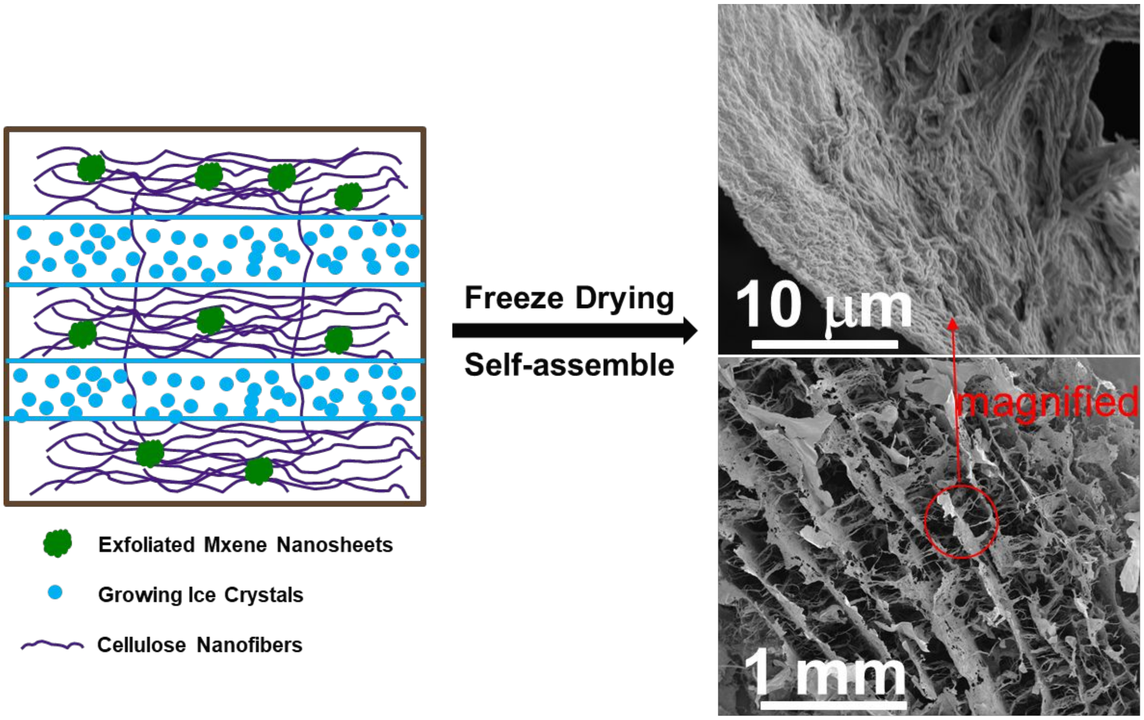

Figure S6. Schematic of formation mechanism of the “accordion-like” cellulose nanofibers / Mxene composite aerogels.

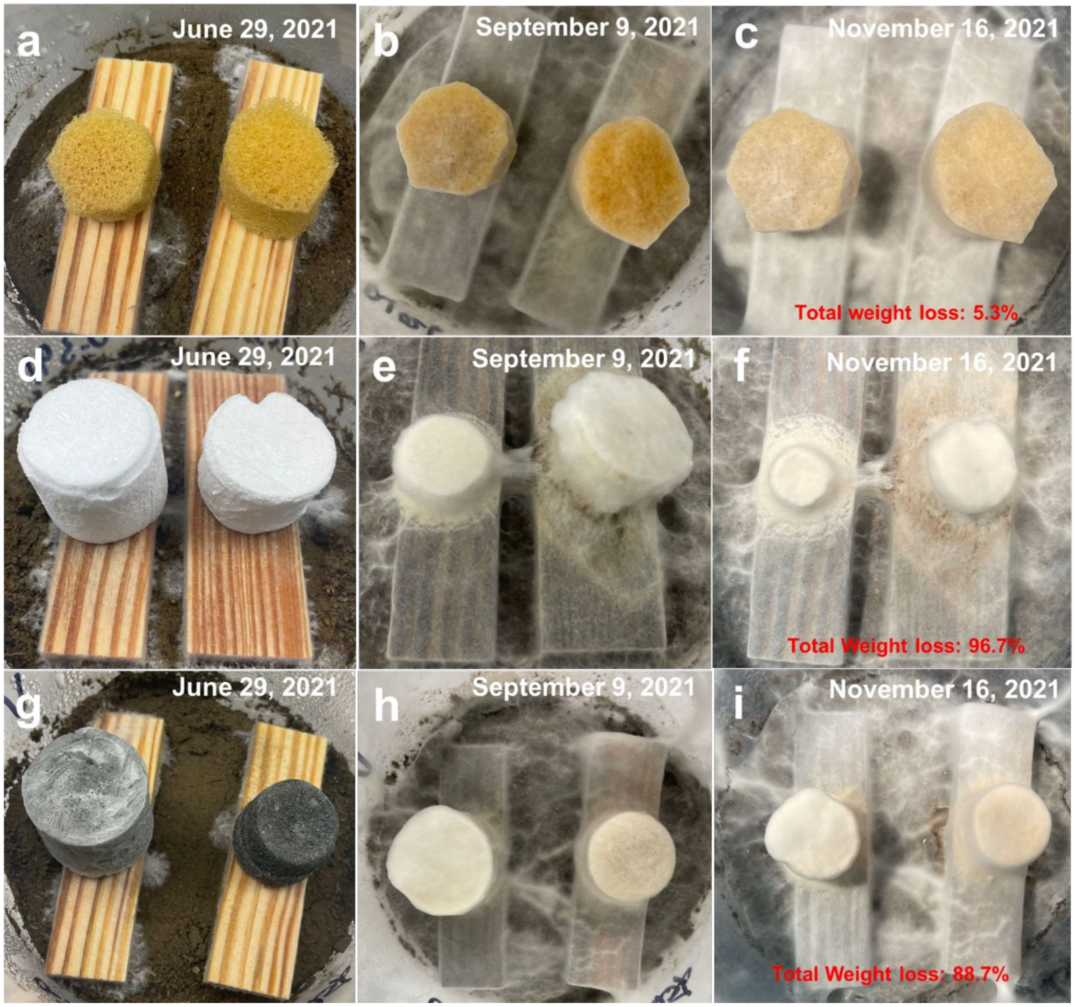

Figure S7. Biodegradability testing of CNF aerogels, CNF/MXene composite aerogels, and synthetic polymer sponges. Digital images of synthetic polymer sponges, pure CNF aerogels and prepared CNF/MXene composite aerogels at original state (a, b, c), biodegraded for 70 days (d, e, f), and biodegraded for 140 days (g, h, i).

Table S1. Summary of performance characteristics comparison of various materials.

Sensing materials	Sensitivity	Response time	Ref.
**CNF/MXene Composite Aerogels**	**3.13** kPa^{-1}	**60 ms**	This work
MXene/PVA sponge	147 kPa^{-1}	138 ms	[1]
MXene textile	3.844 kPa^{-1}	26 ms	[2]
MXene/bacterial cellulose aerogel	12.5 kPa^{-1}	167 ms	[3]
MXene sphere/reduced graphene aerogel	61 kPa^{-1}	232 ms	[4]
TPU/cotton fabric (carbonized)	74.80 kPa^{-1}		[5]
CB/CMC/paper	4.3 kPa^{-1}	240 ms	[6]
rGO/AgNW/cotton fabric	4.23 kPa^{-1}	220 ms	[7]
rGO/cotton fiber	5.8 kPa^{-1}	29.5 ms	[8]
Graphite/paper	27 kPa^{-1}	360 ms	[9]

References

1. Jiang C, Li X, Yao Y, et al. A multifunctional and highly flexible triboelectric nanogenerator based on MXene-enabled porous film integrated with laser-induced graphene electrode. *Nano Energy*. 2019;66:104121.
2. Li T, Chen L, Yang X, et al. A flexible pressure sensor based on an MXene–textile network structure. *Journal of Materials Chemistry C*. 2019;7(4):1022-1027.
3. Chen Z, Hu Y, Zhuo H, et al. Compressible, elastic, and pressure-sensitive carbon aerogels derived from 2D titanium carbide nanosheets and bacterial cellulose for wearable sensors. *Chemistry of Materials*. 2019;31(9):3301-3312.
4. Zhu M, Yue Y, Cheng Y, et al. Hollow MXene sphere/reduced graphene aerogel composites for piezoresistive sensor with ultra‐high sensitivity. *Advanced electronic materials*. 2020;6(2):1901064.
5. Chang S, Li J, He Y, Liu H, Cheng B. A high-sensitivity and low-hysteresis flexible pressure sensor based on carbonized cotton fabric. *Sensors and Actuators A: Physical*. 2019;294:45-53.
6. Liu H, Jiang H, Du F, Zhang D, Li Z, Zhou H. Flexible and degradable paper-based strain sensor with low cost. *ACS Sustainable Chemistry & Engineering*. 2017;5(11):10538-10543.
7. Cao M, Wang M, Li L, Qiu H, Padhiar MA, Yang Z. Wearable rGO-Ag NW@ cotton fiber piezoresistive sensor based on the fast charge transport channel provided by Ag nanowire. *Nano Energy*. 2018;50:528-535.

8. Wei Y, Chen S, Dong X, Lin Y, Liu L. Flexible piezoresistive sensors based on "dynamic bridging effect" of silver nanowires toward graphene. *Carbon*. 2017;113:395-403.
9. Liu H, Xiang H, Ma Y, et al. Flexible, degradable, and cost-effective strain sensor fabricated by a scalable papermaking procedure. *ACS Sustainable Chemistry & Engineering*. 2018;6(11):15749-15755.
